# Understanding AI adoption in healthcare organisations through interacting technological, organisational, and environmental conditions

**DOI:** 10.1108/JHOM-03-2026-0364

**Published:** 2026-09-29

**Authors:** Zaher Ali, Verma Prikshat, Janabelle Abd El Aziz, Didier Vinot

**Affiliations:** ESDES Lyon Business School, Lyon Catholic University, Lyon, France; School of Business and Law, Central Queensland University – Melbourne Campus, Melbourne, Australia; Burgundy School of Business, CEREN EA 7477, Université Bourgogne Europe, Dijon, France; Magellan Research Group EA3713, Chaire Valeurs du Soin, Iaelyon School of Management, Université Jean Moulin Lyon 3, Lyon, France

**Keywords:** AI adoption, Healthcare organisations, TOE framework, Interaction patterns, Qualitative research

## Abstract

**Purpose:**

Artificial intelligence (AI) is widely recognised as a transformative force in healthcare, yet its organisational adoption remains uneven and fragmented across contexts. Existing studies have identified technological, organisational, and environmental conditions influencing AI adoption, but these factors are often examined independently, resulting in inconsistent and inconclusive findings. This study addresses this gap by examining how organisational AI implementation and sustained-use decisions emerge through the interaction of these conditions rather than through isolated effects. Drawing on the Technology–Organisation–Environment (TOE) framework, the study aims to develop a more integrated and context-sensitive understanding of AI adoption in healthcare organisations operating across diverse institutional and regulatory environments.

**Design/methodology/approach:**

The study adopts an inductive qualitative research design grounded in the TOE framework. Data were collected through semi-structured interviews with 29 healthcare experts actively involved in AI adoption across multiple national contexts. These participants represented a range of roles, including clinical leaders, technology specialists, and policy stakeholders, ensuring diverse perspectives on adoption processes. The data were analysed using a Gioia-inspired methodology, enabling systematic identification of first-order concepts, second-order themes, and aggregate dimensions. This approach facilitated the development of a process-oriented and configurational understanding of how technological, organisational, and environmental conditions interact to influence organisational AI implementation and sustained use.

**Findings:**

The analysis identifies six key dimensions influencing AI adoption. Environmental conditions include risk allocation ambiguity and national digital scaffolding. Organisational conditions comprise economic alignment and capability orchestration. Technological conditions involve epistemic transparency and data robustness. A pattern-oriented synthesis reveals that AI adoption emerges through recurring interaction patterns across these dimensions. The findings demonstrate that adoption is not driven by isolated factors such as performance expectations, organisational readiness, or regulatory pressure alone. Instead, technological attributes become consequential only when aligned with organisational capabilities and regulatory accountability, highlighting the fundamentally interactional nature of AI adoption in healthcare contexts.

**Research limitations/implications:**

This study has several limitations. First, the qualitative design and sample of 29 experts support analytical rather than statistical generalisation across diverse healthcare settings. Second, the findings rely on participant perceptions, which may introduce subjectivity and potential bias. Third, although the multi-country sample adds richness, it may obscure important country-specific regulatory and infrastructural differences. Fourth, the cross-sectional design does not capture how AI adoption evolves over time. Finally, the study focuses primarily on expert-level perspectives, with limited representation of frontline healthcare professionals. Future research should adopt longitudinal and mixed-method approaches to examine these interaction patterns across larger and more diverse samples and explore context-specific variations in greater depth.

**Practical implications:**

The findings offer important implications for healthcare managers, policymakers, and technology providers. Organisations should move beyond focusing on individual adoption drivers and instead prioritise alignment across technological capabilities, organisational readiness, and regulatory frameworks. Policymakers should address risk allocation ambiguity and strengthen national digital infrastructures to support AI integration. Healthcare leaders should invest in capability orchestration, ensuring that technical expertise, clinical knowledge, and organisational processes are effectively integrated. Technology developers should enhance epistemic transparency and data robustness to build trust and usability. Overall, a coordinated and system-level approach is essential for enabling effective and sustainable AI adoption in healthcare organisations.

**Social implications:**

AI adoption in healthcare has significant implications for patient outcomes, equity, and system sustainability. By highlighting the importance of interactions across technological, organisational, and environmental conditions, this study provides insights into conditions that may support more responsible and effective organisational AI implementation. Greater attention to these interacting conditions may help healthcare organisations realise potential benefits for clinical practice and patient care while managing unintended risks associated with misaligned systems.

**Originality/value:**

The originality of this study lies in its interactional and process-oriented perspective, moving beyond static models of adoption. It offers a novel explanation for fragmented adoption patterns and provides a comprehensive framework for understanding AI integration in complex healthcare settings.

## 1. Introduction

Artificial Intelligence (AI) is widely expected to transform healthcare by improving diagnostic accuracy, treatment planning, and clinical decision making, through predictive analytics and machine learning-based clinical decision-support systems (Alowais *et al.*, 2023; Khanna *et al.*, 2022; Perivolaris *et al.*, 2024). Despite these anticipated benefits, its adoption into healthcare organisations remains slow, uneven, and markedly more complex than in many other sectors (Davenport and Glaser, 2022; Nair *et al.*, 2024; Schulz *et al.*, 2023). This complexity stems from the high-stakes nature of healthcare delivery, where clinical accountability, ethical sensitivity, regulatory scrutiny, and professional autonomy intersect with rapid technological change. Despite continued advances in AI, including the emergence of generative AI, the adoption of predictive analytics systems in hospitals has largely remained confined to administrative and narrowly defined clinical support functions, falling short of their broader transformative potential (Irgang *et al.*, 2025). In this study, artificial intelligence (AI) refers to data-driven computational systems that use machine-learning algorithms to generate predictions, classifications, or recommendations that can support healthcare-related decision-making. Clinical predictive analytics models learn patterns from historical data to generate predictions or classifications that support tasks such as diagnosis, risk estimation, and clinical decision-making (Beam and Kohane, 2018; Topol, 2019). Accordingly, this study focuses specifically on predictive analytics and machine-learning-based clinical decision-support systems, rather than generative AI, to ensure conceptual consistency across the study period and maintain analytical coherence.

Existing research attributes the slow uptake of AI in healthcare to a constellation of technological, organisational, and environmental factors (Irgang *et al.*, 2025; Yang *et al.*, 2022). At the technological level, concerns surrounding explainability, interpretability, reliability remain central, particularly given the ethical risks associated with algorithmic bias, hallucinations, and data drift in clinical contexts (Cutillo *et al.*, 2020; Jongsma *et al.*, 2024; Pavuluri *et al.*, 2024). These issues directly influence physicians' trust in AI systems and their willingness to integrate such tools into clinical practice (NHS, 2022). At the organisational level, factors such as hospital size, financial constraints, digital infrastructure, and access to specialised talent have been shown to influence adoption trajectories, though their effects are neither uniform nor consistently positive across contexts (Yang *et al.*, 2022). At the environmental level, regulatory frameworks and institutional pressures play a decisive yet contested role, as healthcare organisations must navigate evolving legal, ethical, and policy landscapes while attempting to keep pace with rapid AI innovation (Davenport and Glaser, 2022; Warraich *et al.*, 2025).

Although prior research has identified numerous determinants of AI adoption in healthcare, their influence remains theoretically inconsistent and empirically contested (Davenport and Kalakota, 2019; Greenhalgh *et al.*, 2017). Regulatory and ethical frameworks are alternately portrayed as legitimising mechanisms that enhance trust and facilitate diffusion (European Commission, 2020; WHO, 2021) and as sources of uncertainty, liability risk, and compliance burden that constrain organisational willingness to adopt AI technologies (WHO, 2021). Similarly, organisational enablers such as leadership support, readiness, and staff competencies are often identified as critical drivers of AI adoption (Alami *et al.*, 2020; Jiang *et al.*, 2017), yet other studies report that these factors fail to translate into meaningful use when AI systems conflict with professional autonomy or clinical values (Davenport and Kalakota, 2019; Greenhalgh *et al.*, 2017). At the technological level, predictive accuracy and performance reliability are frequently emphasised as prerequisites for adoption (Davenport and Kalakota, 2019; Reddy *et al.*, 2020), while other research demonstrates that opaque or poorly interpretable models undermine trust and limit clinical integration even when technical performance is high (Grote and Berens, 2020; London, 2019; Price *et al.*, 2019; Vayena *et al.*, 2018).

Beyond theoretical inconsistency, these contradictions also manifest in practice. Healthcare organisations frequently invest in AI technologies that meet regulatory or technical benchmarks yet remain underutilised or resisted in clinical settings, revealing a disconnect between formal adoption decisions and everyday use. Conversely, clinicians may express strong interest in AI tools that demonstrate clinical value but face organisational or regulatory constraints that prevent implementation.

Taken together, these theoretical and practical contradictions point to a common underlying limitation in the existing literature. Although prior studies identify a wide range of technological, organisational, and environmental determinants of AI adoption in healthcare, these factors are predominantly examined as independent drivers rather than as interdependent and mutually conditioning forces. Consequently, the same determinant may facilitate adoption in one organisational context yet constrain it in another because its influence depends on how it interacts with organisational capabilities and environmental conditions. This fragmented analytical approach limits explanatory power and obscures how adoption decisions are actually formed and enacted within healthcare organisations, where formal approval, implementation, and clinical use often diverge.

Accordingly, this exploratory study employs a qualitative research design based on semi-structured interviews with 29 healthcare experts who hold direct responsibilities for AI adoption and integration within diverse organisational contexts. The study addresses the following research question:

How do technological, organisational, and environmental conditions individually and collectively influence organisational decisions regarding AI implementation and sustained use in healthcare organisations?

To address this research question, the study adopts the Technology-Organisation-Environment (TOE) framework, offering a robust and integrative lens to examine AI adoption as an organisational phenomenon emerging from the interaction of technological, organisational, and environmental forces (Hughes *et al.*, 2026). TOE is particularly well suited to healthcare settings, where technological characteristics, organisational capabilities, and environmental constraints jointly influence adoption decisions.

This study makes three key contributions to the literature on AI adoption in healthcare. First, it contributes conceptually by extending determinant-based explanations of AI adoption by suggesting how technological, organisational, and environmental conditions interact to inform and condition adoption decisions within healthcare organisations. Rather than treating these conditions as independent influences, the study suggests how adoption decisions emerge through recurring interaction patterns across organisational contexts.

Second, the study contributes empirically by providing in-depth qualitative insights from healthcare experts directly involved in AI adoption and integration. These insights illuminate the often-overlooked gap between formal adoption decisions and actual clinical use, revealing how organisational priorities, technological characteristics, and environmental constraints jointly influence how AI is adopted in practice. By foregrounding lived organisational experiences, the study develops a richer understanding of AI adoption as a dynamic and context-dependent organisational process rather than a binary implementation outcome.

Third, the study contributes to practice by identifying recurring interaction patterns among technological characteristics, organisational conditions, and environmental constraints that shape AI adoption in healthcare settings. These insights provide healthcare leaders, policymakers, and technology developers with a more realistic basis for evaluating organisational readiness by illustrating how adoption challenges and opportunities arise from the alignment, or misalignment, of multiple interacting conditions rather than isolated determinants.

## 2. Literature review

### 2.1 Artificial intelligence adoption in healthcare organisations

AI has been widely promoted as a transformative technology capable of improving diagnostic accuracy, operational efficiency, and clinical decision-making in healthcare systems. Despite these expectations, empirical evidence shows that AI adoption in healthcare organisations remains uneven, fragmented, and slower than anticipated, particularly compared to other data-intensive sectors (Davenport and Glaser, 2022; Greenhalgh *et al.*, 2017).

Existing research suggests that AI adoption in healthcare results from the interplay of technical constraints, professional norms, regulatory oversight, and ethical considerations. Healthcare contexts are characterised by high-stakes decision-making, strong professional autonomy, and stringent accountability requirements, which amplify perceived risks associated with AI use (Amann *et al.*, 2020; Cresswell and Sheikh, 2013). As a result, adoption decisions are rarely binary and instead involve careful negotiation over where and how AI systems can be safely deployed. Consequently, AI adoption is rarely a discrete implementation decision but rather an iterative organisational process involving continuous negotiation over where, when, and under what conditions AI systems can be safely and legitimately integrated into clinical practice.

### 2.2 The technology-organisation–environment (TOE) framework and AI adoption

Individual-level acceptance models, such as the Technology Acceptance Model (TAM) and the Unified Theory of Acceptance and Use of Technology (UTAUT), explain technology acceptance primarily through individual perceptions and behavioural intentions, including perceived usefulness, perceived ease of use, social influence, and willingness to use a technology. These models provide important insights into clinicians' acceptance and use of AI but address a different level of analysis from the organisational adoption decisions examined in this study. Here, the focal outcome is organisational adoption, which involves collective decisions concerning whether, how, and under what conditions healthcare organisations evaluate, implement, and sustain AI technologies. Individual-level responses are not assumed to be irrelevant to this process. Indeed, clinicians' trust, willingness to engage with AI, and interpretive effort emerged in participants' accounts. However, these individual-level responses are examined insofar as they influence organisational implementation and sustained use, rather than as outcomes of individual technology acceptance.

Accordingly, drawing on research on organisational technology adoption in healthcare and the TOE framework (Duus *et al.*, 2026; Nair *et al.*, 2025; Tornatzky and Fleischer, 1990), this study conceptualises organisational AI adoption as the process through which healthcare organisations evaluate, commit to, implement, and sustain AI technologies within clinical and organisational systems. The focal decisions therefore concern whether, how, and under what conditions an organisation proceeds with AI implementation and sustained use. This organisational-level conceptualisation distinguishes the study's outcome from individual clinicians' acceptance or behavioural intention, while recognising that individual responses may influence organisational implementation decisions.

The TOE framework provides a robust lens for examining these dynamics by accounting for the interaction between technological characteristics, organisational capabilities, and environmental conditions (Duus *et al.*, 2026). Prior healthcare studies applying TOE demonstrate that AI adoption is context-dependent and shaped by the alignment of these dimensions. For example, technological risks and regulatory uncertainty may constrain adoption even when organisational readiness is high (Yang *et al.*, 2022), while organisational and environmental support jointly influence digital health adoption trajectories (Jeilani and Hussein, 2025; Nevi *et al.*, 2025). Accordingly, the TOE framework provides an appropriate foundation for examining AI adoption as an organisational phenomenon shaped by multiple interacting conditions rather than isolated determinants. Beyond AI, the TOE framework has also been successfully applied to explain organisational adoption of other healthcare digital innovations, including blockchain technologies, demonstrating its continued relevance for understanding complex technology adoption processes (Dbesan *et al.*, 2025).

#### 2.2.1 Configurational and relational perspectives on organisational adoption

While the TOE framework provides a well-established structure for examining the technological, organisational, and environmental determinants of AI adoption, it does not explicitly theorise the configurational or interactional relationships among these dimensions. Set-theoretic approaches, particularly Ragin (2008), argue that organisational outcomes emerge through conjunctural and equifinal combinations of conditions rather than independent causal effects. Similarly, configurational perspectives advanced by Meyer *et al.* (1993) and Greenwood *et al.* (2011) conceptualise organisations as configurations of structures and practices that are shaped by their institutional environments. Extending this perspective, Orlikowski's (2000) process-relational view and Actor-Network Theory (Callon, 1986; Latour, 1987; Law, 1992) emphasise that technology adoption emerges through ongoing interactions among human actors, technologies, and institutional arrangements rather than through the influence of isolated factors.

Although these perspectives provide a strong theoretical foundation for understanding how organisational outcomes emerge through interacting conditions, they offer limited empirical specification of how such interactions unfold in the context of AI adoption within healthcare organisations. Accordingly, this study adopts the TOE framework as its primary analytical lens, while drawing on configurational and relational perspectives to inform the interpretation of how technological, organisational, and environmental conditions interact during AI adoption.

The purpose of this study is therefore not to establish that organisational outcomes emerge through interacting conditions, as this premise is already well established within configurational and relational scholarship. Rather, the contribution lies in identifying the specific technological, organisational, and environmental interaction patterns through which AI adoption emerges in healthcare organisations and explaining how these patterns unfold within a highly regulated, professionally governed, and risk-sensitive context. In doing so, the study extends configurational thinking within the healthcare AI adoption literature by providing an empirically grounded explanation of how recurring interaction patterns influence organisational adoption decisions.

While configurational approaches, including set-theoretic methods such as fsQCA, are well suited to identifying combinations of conditions associated with organisational outcomes, they generally provide limited insight into the organisational processes through which these configurations are enacted (Ragin, 2008; Misangyi *et al.*, 2017). By contrast, abductive qualitative inquiry seeks to explain how such configurations emerge, are negotiated, and evolve through organisational action and interaction (Langley *et al.*, 2013; Gioia *et al.*, 2013; Orlikowski, 2000). Accordingly, the present study complements configurational research by explaining how technological, organisational, and environmental conditions interact in practice as healthcare organisations negotiate AI adoption under conditions of professional accountability, regulatory uncertainty, and organisational complexity.

#### 2.2.2 Technological context and AI adoption in healthcare

AI adoption in healthcare is constrained by fundamental challenges related to both data and model characteristics (Dwivedi *et al.*, 2023). Bias in datasets remains a persistent concern, reflecting underlying social and demographic inequalities and limiting fairness in AI outputs (Geis *et al.*, 2019). In addition, limited generalisability across populations, settings, and time reduces the reliability of AI systems in diverse clinical environments (Browne, 2023; Duckworth *et al.*, 2021; Seyyed-Kalantari *et al.*, 2021).

Model-related characteristics, including explainability, interpretability, and opacity, further influence adoption. In high-risk clinical settings, the inability to understand how AI systems generate outputs undermines trust and limits their integration into practice (Liu *et al.*, 2025; Rosenbacke *et al.*, 2024).These challenges are compounded by trade-offs between key technological attributes. Studies highlight tensions between accuracy and transparency, generalisability, and scalability, indicating that improvements in one dimension may come at the expense of others (Hunsicker *et al.*, 2025; Yang *et al.*, 2022). Such trade-offs complicate adoption decisions by requiring organisations to balance competing technological priorities.Thus, organisations must balance competing technological priorities when evaluating the suitability of AI systems for clinical implementation.

#### 2.2.3 Organisational context and AI adoption in healthcare

Organisational factors influencing AI adoption operate at both workforce and structural levels. At the workforce level, AI adoption depends on clinicians' skills, awareness, and engagement, which are developed through training, literacy initiatives, and exposure to AI technologies (Hassan *et al.*, 2024; Schuitmaker *et al.*, 2025). Greater awareness has been associated with increased acceptance and willingness to use AI in clinical settings (Hemphill *et al.*, 2023; Vijayakumar *et al.*, 2023). At the same time, effective integration of AI into workflows and collaboration between clinicians and AI systems are critical for translating adoption into practice (Brennan and Kirby, 2022; Chew and Achananuparp, 2022).

At a broader level, organisational readiness plays a central role in shaping adoption outcomes. Factors such as strategic alignment, financial resources, technological infrastructure, and organisational culture influence the ability to initiate and sustain AI initiatives (Lokuge *et al.*, 2019; Nguyen *et al.*, 2019). Leadership-driven prioritisation and resource allocation further determine which AI projects progress within organisations (Strohm *et al.*, 2020; Sun and Medaglia, 2019; Ismael *et al.*, 2025). Additionally, access to specialised expertise and multidisciplinary collaboration, particularly involving clinical and technical professionals, is critical for successful implementation (Hickman *et al.*, 2021; Victor Mugabe, 2021).

#### 2.2.4 Environmental context and AI adoption in healthcare

At the environmental level, AI adoption in healthcare is conditioned by regulatory, legal, and governance conditions. Regulatory uncertainty and fragmented policy environments are widely identified as barriers, particularly where inconsistencies exist between legal frameworks and ethical standards (Churi *et al.*, 2022; Olawade *et al.*, 2025; Wolff *et al.*, 2021). The absence of clear enforcement mechanisms further contributes to hesitation among organisations and professionals.

Governance challenges are closely linked to issues of accountability and liability. Healthcare professionals are subject to strict regulatory obligations for clinical decisions, whereas AI developers operate under less formalised accountability structures (Smith, 2021). This asymmetry creates uncertainty regarding responsibility for AI-assisted outcomes, particularly in the absence of established legal precedents (Bottomley and Thaldar, 2023).

In response, organisations increasingly establish internal governance mechanisms that bring together clinical, technical, and policy stakeholders to oversee AI implementation. These arrangements reflect the need to manage risk, ensure accountability, and align AI use with broader institutional and societal expectations (Hassan *et al.*, 2024; Hemphill *et al.*, 2023; Abdulmuhsin *et al.*, 2026).

## 3. Methodology

### 3.1 Research design and philosophical positioning

This study adopts an exploratory qualitative research design to investigate AI adoption in healthcare organisations. Prior research highlights that complex, context-dependent organisational phenomena such as AI adoption require interpretive depth and contextual sensitivity (Creswell and Creswell, 2022; Greenhalgh *et al.*, 2017). Consistent with an interpretivist orientation, the study seeks to develop analytically grounded and contextually informed explanations of AI adoption rather than statistically generalisable findings (Lincoln and Guba, 1985). The emphasis is therefore placed on understanding how organisational actors interpret, negotiate, and enact AI adoption within their institutional contexts.

### 3.2 Research approach and analytical framework

The study is informed by the TOE framework, which explains organisational technology adoption through technological, organisational, and environmental conditions (Baker, 2012; Tornatzky and Fleischer, 1990). Rather than serving as a predetermined coding framework, TOE functioned as a sensitising framework that guided the design of the interview protocol and the early stages of analysis while allowing concepts and themes to emerge through iterative engagement with the empirical data (Miles *et al.*, 2020). Accordingly, the study adopts an abductive, theory-sensitised analytical approach in which insights were developed through continuous movement between empirical observations and existing theoretical explanations.

Building on the initial thematic analysis, the analytical process proceeded to a second-stage interpretive synthesis that examined how technological, organisational, and environmental themes interacted to produce AI adoption decisions, rather than treating these conditions as independent influences (Eisenhardt, 1989; Langley, 1999; Sinkovics, 2018). This second stage focused on identifying recurring interaction patterns across cases that explained how multiple conditions jointly influenced organisational adoption decisions.

To support systematic theory development, the study follows the Gioia methodology, enabling transparent progression from informant-centric first-order concepts to researcher-centric second-order themes, and finally to aggregate dimensions (Gioia *et al.*, 2013). The resulting Gioia data structures provide an explicit audit trail linking participants' accounts to the conceptual dimensions developed through the abductive analytical process, thereby enhancing the transparency and credibility of the study.

### 3.3 Sampling strategy and participants

A purposive sampling strategy was used to identify information-rich participants with direct experience of AI adoption and implementation within healthcare contexts. The final sample comprised 29 participants drawn from multiple countries and healthcare systems (See Table 1 for more details). Participants were selected based on their involvement in organisational-level AI initiatives. To be eligible, participants were required to be practising clinicians and demonstrate active involvement in AI adoption through leadership roles, responsibilities for AI or digital transformation initiatives, or recognised expertise evidenced through scholarly, technical, or policy contributions. Potential participants were identified through hospital websites, professional healthcare AI networks, academic publications, LinkedIn profiles, and referrals from participants. Individuals were invited when they met the study's inclusion criteria and had direct experience or responsibility related to AI adoption or implementation within healthcare organisations. This purposive recruitment strategy was intended to identify information-rich participants with first-hand knowledge of organisational decision-making concerning AI implementation. A total of 60 participants were invited. While 30 agreed to be interviewed, the remaining individuals, 11 declined participation, primarily due to time or scheduling constraints, while 19 did not respond to the invitation. Following data verification, one interview was excluded because the participant did not meet the study's inclusion criteria, having represented a predominantly AI-vendor perspective without direct responsibility for AI implementation within a healthcare organisation. The final analytical sample therefore comprised 29 participants.

**Table 1:** Participant demographics and professional information

S.NO	Professional role	Organisation type	Country	Organisation size	Years of experience
1	Medical Doctor-Global Chief Medical Information officer	Academic Medical Education Institutions	US	Large-scale	20–25 years
2	Medical Doctor-Clinical Research Director-Co-director of Sana	Academic Medical Education Institutions	US	Large-scale	25–30 years
3	Medical Doctor-Chief Medical Information officer	University Hospitals	US	Large-scale	40 years
4	Medical Doctor-Chief Medical Information Officer	University Hospital	US	Large-scale	25 years
5	Medical Doctor-Chief Medical Information Officer	University Hospitals	US	Large-scale	25 years
6	Medical Doctor-Medical Director-Chief of Division of Critical Care	University Hospitals	US	Large-scale	15 years
7	Medical Doctor-Founder and Director- Augmented imaging/Artificial intelligence Data Analytics (AiDA) laboratory	University Hospitals	US	Large-scale	12 years
8	Medical Doctor-EpicCare Ambulatory Solution Engineer and Physician	University Hospitals	US	Large-scale	14 years
9	Medical Doctor-Chief Innovation officer and Chief Medical Officer	University Hospitals	US	Small	15–18 years
10	Medical Doctor-Chief Executive Officer	University Hospitals	France	Large-scale	18 years
11	Medical Doctor-Deputy Chief-Diagnostic and Interventional Radiology	University Hospitals	France	Large-scale	19 years
12	Medical Doctor- Head of Department & Head of Division – Vice-Chair of the Medical Board	University Hospitals	France	Large-scale	19–20 years
13	Medical Doctor-Clinical Innovation Lead	University Hospitals	France	Large-scale	19–20 years
14	Medical Doctor-President of the Lyon College of Orthopaedics	University Hospitals	France	Large-scale	25 years
15	Medical Doctor-President CAOS-France, Director RHU ReBone et FHU Plan&Go	University Hospitals	France	Large-scale	15–16 years
16	Medical Doctor-President of the Ethics Technology Committee	University Hospitals	France	Large-scale	12–15 years
17	Medical Doctor-Founder-Global Medical Strategist - Digital & AI Healthcare Transformation	Health organisation	France	Large-scale	6 years
18	Medical Doctor-Regional office for Europe (AI and technology)	Public Health & International Health Organisation	Netherlands	Large-scale	22–24 years
19	Medical Doctor-Founder of the National eHealth Living Lab-Chair of E-Health Applications	University Hospitals	Netherlands	Large-scale	28 years
20	Medical Doctor-Chief of the “Advanced Medical Imaging-AI	Hospital	Spain	Large-scale	10–12 years
21	Medical Doctor-Consultant-AI in Healthcare	Academic Medical and Clinical Institution	Singapore	Large-scale	10–12 years
22	Medical Doctor-Chief Medical Officer	Academic Medical and Clinical Institution	Singapore	Small	30 years
23	Medical Doctor-Director and Co-founder	Hospital & Health-tech organisation	Romania	Small	25 years
24	Medical Doctor-President and Co-founder	Healthcare Innovation & Clinical Policy Organisation	Romania	Small	20–22 years
25	Medical Doctor-Co-Director-Health Ethics and Technology Governance	Public Health & International Health Organisations	Switzerland	Large-scale	28–30 years
26	Director-Head of AI Collaborations	Healthcare Systems & National Health Services	UK	Large-scale	10–15 years
27	Medical Doctor-AI Program Manager	Healthcare Systems & National Health Services	UK	Large-scale	8–12 years
28	Medical Doctor-Healthcare Data Scientist	Hospital	Estonia	Small	8 years
29	Medical Doctor-AI clinical Success Manager	Hospital & Health-tech organisations	Belgium	Small	10 years

**Source(s):** Authors’ own work

The sample included senior clinical, technical, managerial, and governance professionals across university hospitals, academic medical centres, public health organisations, national health systems, and healthcare technology organisations. While some participants have hybrid roles in healthcare organisations and health-technology companies, they were included based on their organisational responsibilities in healthcare systems rather-than vendor-side activities.

In sum, all the selected participants work at the intersection of clinical practice and organisational decision making. Therefore, their perspective is informed by their direct interaction with AI models and by their experience overseeing and managing the implementation of AI projects. As a result, their perspectives account for clinical practice realities as well as the technical and organisational dimensions, rather than addressing only one of these dimensions.

### 3.4 Cross-country sampling rationale

The inclusion of participants from multiple countries reflects a deliberate design choice aligned with the study's aim of identifying higher-order determinants of AI adoption in healthcare organisations. Restricting the analysis to a single country would risk generating context-bound insights and limit theoretical development. Consistent with qualitative research principles, the study seeks analytical rather than statistical generalization by identifying patterns across diverse contexts (Eisenhardt, 1989; Welch *et al.*, 2011). Cross-country sampling is particularly valuable when examining institutionally embedded technologies, as it enables the identification of shared mechanisms while capturing contextual variation (Greenhalgh *et al.*, 2017; Hennink and Kaiser, 2022).

Although participants represented multiple healthcare systems, the sample was concentrated in the United States and France because these countries provided greater access to participants who satisfied the study's inclusion criteria. Consequently, country selection was driven by the theoretical relevance and organisational roles of participants rather than by an intention to achieve geographical representativeness.

Accordingly, this study does not seek to compare countries or identify country-specific adoption patterns. Instead, the cross-country design was intended to strengthen the analytical robustness of the findings by identifying recurring technological, organisational, and environmental conditions, together with their interaction patterns, across diverse healthcare contexts. The concentration of participants in the United States and France is acknowledged as a limitation and is considered in the interpretation of the study's transferability rather than its analytical validity.

### 3.5 Data collection

Data were collected through semi-structured, in-depth interviews conducted between September 2021 and May 2025. Although this period coincided with substantial advances in AI technologies, including the emergence of generative AI, the study focused exclusively on predictive analytics and machine learning-based clinical decision-support systems. These technologies evolved incrementally throughout the data collection period without fundamental changes to their underlying organisational adoption processes, thereby ensuring conceptual consistency across the study. Consequently, interview discussions relating specifically to generative AI were considered beyond the scope of this research. The interview protocol remained consistent throughout the data collection period to ensure comparability across participants and over time.

Interviews were conducted either face-to-face or via Zoom, depending on participant availability and location, with an average duration of approximately 45 min. Although participants are drawn from different countries, all interviews were conducted in English. Participants confirmed their ability to communicate fluently in English. Conducting all interviews in a single language eliminated the need for translation during transcription and analysis, thereby reducing the potential loss of conceptual meaning associated with cross-language qualitative research. Therefore, during data collection, participants' accounts have single language without need for translation, which could result in the loss of nuance in qualitative analysis.

The interview guide was informed by the TOE framework and included open-ended questions addressing technological, organisational, and environmental determinants of AI adoption. Consistent with the study's abductive, theory-sensitised approach, the interview questions were designed to encourage participants to describe their experiences freely rather than to confirm predetermined theoretical categories. Probing questions explored issues such as governance, ethics, regulatory uncertainty, and professional accountability, all of which are recognised as central to AI adoption in healthcare (WHO, 2021). With participants' informed consent, all interviews were audio-recorded and transcribed verbatim to ensure the accuracy and completeness of the qualitative dataset. To minimise interviewer bias and avoid leading participants towards predetermined explanations, questions were deliberately phrased in an open-ended and non-directive manner, allowing participants to introduce issues they considered most relevant to their organisational experiences before follow-up probes were used for clarification.

### 3.6 Data analysis

This study adopted an abductive, theory-sensitised qualitative approach to examine how technological, organisational, and environmental conditions interact to inform AI adoption decisions in healthcare organisations. Data analysis was conducted iteratively, moving between close engagement with participants' accounts and higher-order theoretical interpretation. Initially, first-order concepts and second-order themes were developed inductively from participants' narratives. These emerging concepts were subsequently interpreted through an abductive analytical process that involved continuous movement between the empirical data and existing theoretical perspectives. Accordingly, the aggregate dimensions were not mechanically derived from the Technology-Organisation-Environment (TOE) framework. Instead, they emerged through iterative engagement with the data while being interpreted through a theory-sensitised analytical lens. As a result, the identified dimensions extend and refine existing TOE understandings within the context of healthcare AI adoption rather than simply reproducing its predefined categories.

Throughout the analytical process, coding decisions were discussed iteratively among the research team until consensus was reached regarding first-order concepts, second-order themes, and aggregate dimensions. Consistent with the interpretivist orientation of the Gioia methodology, the analysis prioritised conceptual agreement through collaborative interpretation rather than statistical measures of inter-coder reliability (Gioia *et al.*, 2013).

Given the extended data collection period, special attention was devoted to assessing the potential influence of temporal heterogeneity on the findings. The interviews were conducted between 2021 and 2025, a period characterised by substantial technological, regulatory, and institutional developments within the broader AI landscape. Although the present study focused exclusively on predictive analytics and machine learning-based clinical decision-support systems, rather than generative AI technologies, these broader developments could still have influenced how participants discussed AI adoption in healthcare organisations.

To examine this possibility, the interviews were analysed on a year-by-year basis to assess the stability of both the thematic dimensions and the interaction patterns across the study period. Specifically, transcripts were reviewed chronologically and compared across successive phases of data collection to determine whether new first-order concepts emerged, whether existing second-order themes changed in meaning, and whether the relationships underpinning the interaction patterns varied over time. This temporal comparison did not reveal substantive differences in the conceptual meaning of the identified dimensions or the interaction patterns. Although later interviews reflected increased awareness of evolving AI technologies and regulatory developments, these changes did not alter the underlying organisational mechanisms shaping predictive AI adoption. Consequently, the findings were considered analytically consistent across the study period.

In addition, several participants held hybrid positions spanning healthcare organisations and health technology companies. To minimise potential commercial bias, the analysis prioritised participants' accounts relating specifically to healthcare implementation, organisational governance, and adoption decision making. Throughout coding and interpretation, particular attention was paid to distinguishing healthcare-provider perspectives from potentially supplier-oriented viewpoints, thereby ensuring that the findings reflected organisational adoption experiences rather than vendor interests.

#### 3.6.1 Thematic analysis

An abductive, theory-sensitised thematic analysis was conducted to identify recurring patterns across the dataset (Braun and Clarke, 2006). To assess the potential influence of temporal heterogeneity, the analysis was undertaken on a year-by-year basis, comparing interviews conducted at different stages of the data collection period. This chronological comparison examined whether the meaning, manifestation, or relevance of the emerging concepts, themes, and aggregate dimensions varied over time.

The temporal analysis did not reveal substantive differences in the six aggregate dimensions across the study period. Although interviews conducted in later years occasionally reflected increased awareness of evolving AI technologies and regulatory developments, these references did not alter the conceptual meaning or organisational relevance of the identified dimensions. Accordingly, the dimensions were considered analytically stable throughout the 2021–2025 data collection period.

Interview transcripts were coded to generate first-order concepts using participants' terminology, which were then clustered into research-centric second-order themes which were then synthesised into aggregate dimensions through an iterative abductive process informed, but not predetermined, by the Technology-Organisation-Environment (TOE) framework. This analytical process allowed healthcare-specific mechanisms to emerge from the empirical data while maintaining theoretical sensitivity to existing organisational adoption literature.

Analysis involved constant comparison across interviews, contexts, and years. Qualitative data analysis software supported coding and data management. Data collection and analysis continued until theoretical saturation was reached defined as the point at which additional data no longer generated new insights (Hennink and Kaiser, 2022). Consistent with the study's analytical objectives, saturation was assessed at the level of second-order themes and aggregate dimensions rather than across individual countries or participant categories. Specifically, saturation was considered achieved when additional interviews no longer generated new first-order concepts relating to the technological (epistemic transparency and data robustness), organisational (capability orchestration and economic alignment), and environmental (risk allocation ambiguity and national digital scaffolding) dimensions. Later interviews reinforced and enriched the existing conceptual categories without introducing substantively new themes, providing further evidence of thematic saturation and supporting the analytical coherence of the dataset across the study period.

#### 3.6.2 Second-stage interpretive pattern-based synthesis

Building on the thematic analysis, a second-stage interpretive, pattern-based synthesis examined how technological, organisational, and environmental themes interacted to inform AI adoption decisions. This stage focused on identifying recurring interaction patterns across themes rather than treating them as independent influences (Eisenhardt, 1989; Langley, 1999). Consistent with the findings of the temporal analysis, the interaction patterns remained analytically stable across the data collection period. Although later interviews occasionally reflected changes in the broader AI landscape, no substantive differences were observed in the relationships underpinning the identified interaction patterns between earlier and later interviews.

Themes were systematically compared across participants and organisational contexts to identify recurrent combinations of technological, organisational, and environmental conditions that consistently reinforced, constrained, or moderated one another. Using a pattern-matching logic, these interactions were interpreted as context-dependent empirical regularities rather than deterministic causal relationships (Sinkovics, 2018). Some interaction patterns were explicitly articulated by participants through descriptions of interdependent organisational conditions, whereas others emerged through cross-case comparison and abductive interpretation of recurring relationships across the dataset.

Accordingly, this stage did not seek to develop a formal configurational model or test causal propositions. Instead, it aimed to explain how combinations of interacting conditions shape organisational AI adoption within the complex, highly regulated context of healthcare. The resulting interaction patterns are presented in Section 5.

Following identification of the five interaction patterns, we re-examined the coded interview material to document the distribution of supporting accounts across participants and contexts, distinguishing explicitly articulated relationships from those identified through cross-case abductive analysis.

### 3.7 Researcher reflexivity

This study was conducted by researchers with multidisciplinary backgrounds in healthcare, management, and human resource management, whose research focuses on digital innovation, organisational transformation, and artificial intelligence. This disciplinary diversity enabled the phenomenon of AI adoption to be examined from complementary perspectives while reducing reliance on any single theoretical or professional lens.

The researchers had no prior professional or personal relationships with the participants and did not hold operational or managerial positions within the participating healthcare organisations, thereby reducing the potential influence of pre-existing relationships on data collection and interpretation. Recognising that qualitative analysis is inherently interpretive, the research team remained reflexive throughout the study by continuously considering how their disciplinary backgrounds, assumptions, and theoretical perspectives might influence the interpretation of participants' accounts.

To minimise these influences, the interview guide employed open-ended questions that encouraged participants to describe their experiences in their own terms rather than respond to predetermined expectations. During analysis, the researchers engaged in iterative discussions, collaborative coding, and constant comparison of interpretations to challenge emerging assumptions and ensure that conceptual development remained grounded in participants' accounts rather than researchers' preconceptions. This reflexive process strengthened the credibility, transparency, and confirmability of the analytical findings.

### 3.8 Trustworthiness

The study followed Lincoln and Guba's (1985) criteria to ensure methodological rigor. Credibility was supported through iterative analysis, including year-by-year comparison of interviews and close engagement with the data. Transferability was addressed by providing contextual detail to enable assessment of applicability across settings. Dependability was ensured through collaborative coding and documentation of analytical decisions (Miles *et al.*, 2014). Confirmability was strengthened through investigator triangulation, with independent review of coding and interpretations to maintain analytical transparency. The study was conducted in compliance with the ethical standards of Université Jean Moulin Lyon 3 (Magellan Lab) and received formal institutional ethical approval prior to data collection.

## 4. Results

### 4.1 Organisational factors

The findings indicate that organisational factors constitute central conditions impacting AI implementation within healthcare organisations. Specifically, they determine how resources are allocated, capabilities are mobilised, and strategic priorities are established. Rather than operating as isolated organisational attributes, these factors collectively determine an organisation's capacity to initiate, implement, and sustain AI implementation. Figure 1 shows the Gioia data structure for the two themes followed by their discussion.

**Figure 1:**
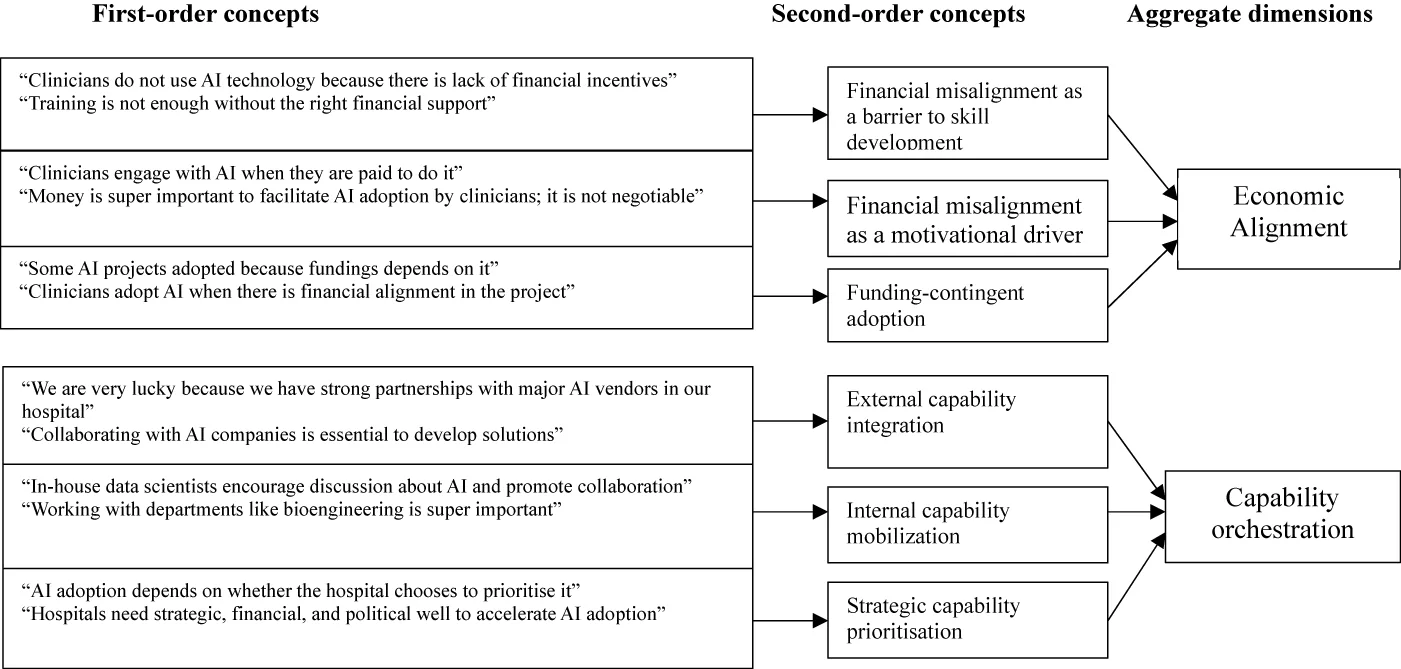
Gioia data structure: organisational conditions shaping AI adoption. Source: Authors’ own work

#### 4.1.1 Economic alignment

Healthcare professionals involved in AI adoption consistently identified economic alignment as a foundational organisational condition enabling AI implementation and sustained use within healthcare organisations. Participants described economic alignment as determining whether time, effort, and resources could be allocated to AI related activities, particularly training, experimentation, and system integration, alongside demanding clinical workloads. Beyond motivating individual engagement, economic alignment also signalled organisational commitment to AI adoption by influencing strategic investment decisions, project prioritisation, and the allocation of implementation resources. In several cases, adoption trajectories were further conditioned by external funding arrangements, demonstrating that organisational investment decisions were closely intertwined with broader environmental funding mechanisms. Consequently, economic alignment emerged not simply as a financial consideration but as an organisational condition that operated through strategic priorities, resource availability, and institutional commitment collectively influenced the pace, scope, and sustainability of AI implementation.

#### 4.1.2 Capability orchestration

Analysis of participants' accounts revealed a recurring pattern centred on how healthcare organisations actively assembled and coordinated resources to enable AI implementation. Across interviews, participants emphasised the importance of maintaining strong relationships with AI vendors, academic institutions, and technology partners, which increased exposure to AI innovations and facilitated collaborative development. These external connections were frequently complemented by internal arrangements, such as clinical data science forums and dedicated AI communities, that enabled knowledge exchange and collective problem solving. In contrast, the absence of in-house AI expertise or limited access to skilled personnel constrained organisations' ability to initiate or sustain AI projects. Participants also highlighted how organisational decisions regarding project selection and resource allocation determined which AI initiatives progressed beyond individual or research-oriented efforts. Taken together, these findings point to an organisational capability through which healthcare organisations deliberately mobilise, integrate, and coordinate internal and external resources to support AI adoption. This organisational condition is conceptualised in this study as *capability orchestration*.

Although capability orchestration shares similarities with broader concepts such as dynamic capabilities and resource orchestration, it is conceptualised more narrowly in this study as the deliberate coordination of clinical expertise, technical knowledge, governance arrangements, and external partnerships specifically to support the implementation, monitoring, and sustained use of AI within healthcare organisations (Sirmon *et al.*, 2011; Teece, 2007).

### 4.2 Environmental factors

The findings indicate that environmental factors constitute critical conditions influencing AI implementation in healthcare by defining the broader institutional, regulatory, and infrastructural conditions within which organisations operate. Figure 2 shows the Gioia data structure for the two themes of environmental conditions shaping AI adoption.

**Figure 2:**
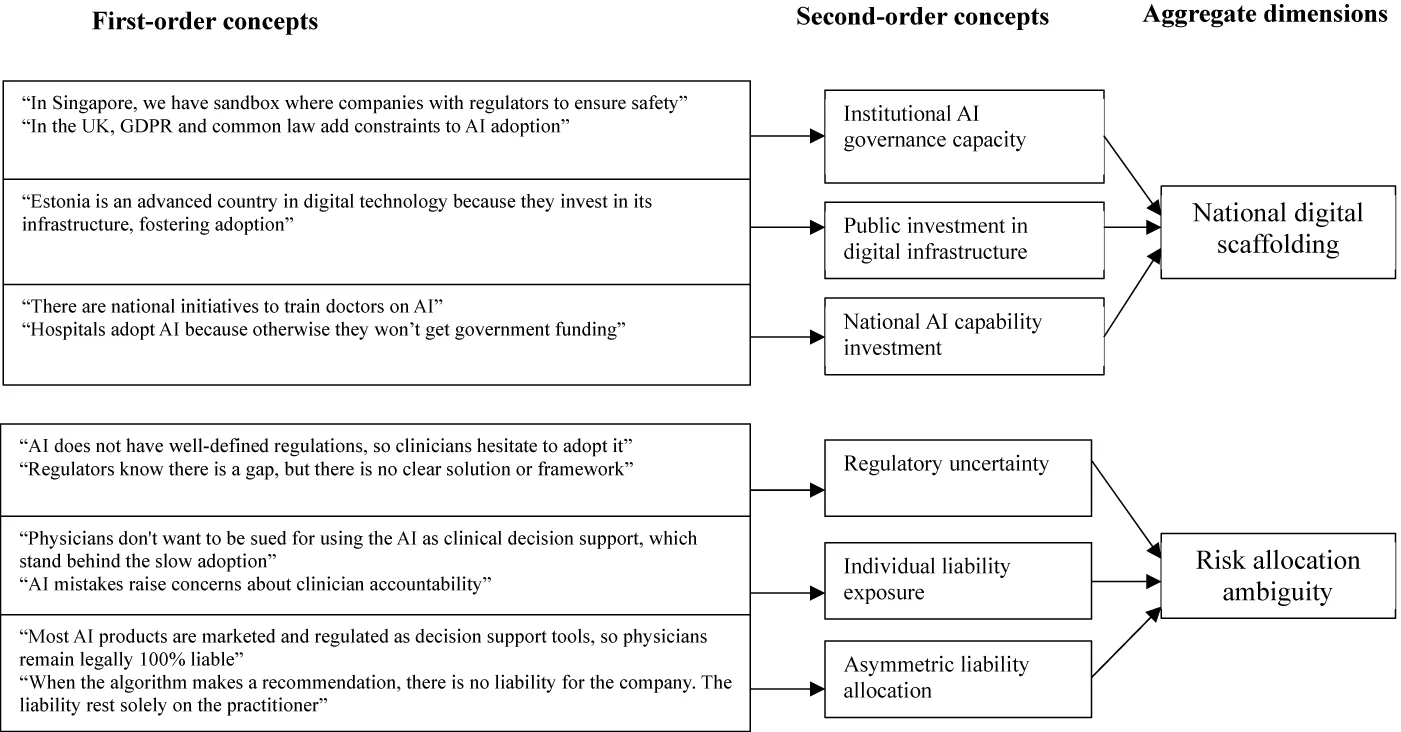
Gioia data structure: environmental conditions shaping AI adoption. Source: Authors’ own work

#### 4.2.1 Risk allocation ambiguity

Analysis of participants' accounts revealed that uncertainty surrounding the allocation of responsibility and accountability among stakeholders constitutes a central environmental condition constraining AI implementation and sustained use in healthcare. Participants consistently pointed to regulatory uncertainty, noting that the absence of clear and harmonised AI regulations created hesitation and safety concerns, particularly in contexts involving multiple stakeholders. This uncertainty was compounded by individual liability exposure, as clinicians remained legally responsible for clinical decisions supported by AI systems, including errors arising from AI outputs. Participants further highlighted asymmetric liability allocation, whereby AI vendors deliberately framed their products as decision-support tools, thereby limiting their own liability while transferring accountability to healthcare professionals.

Collectively, these conditions generated persistent ambiguity regarding responsibility, liability, and professional accountability, discouraging organisational willingness to integrate AI and contributing to cautious, risk-averse engagement with AI technologies within healthcare organisations. Accordingly, this study conceptualises these interrelated conditions as *risk allocation ambiguity*, an environmental mechanism through which uncertainty surrounding legal and professional responsibility constrains organisational AI implementation.

#### 4.2.2 National digital scaffolding

Analysis of participants' accounts indicates that national-level digital conditions constitute an important environmental condition impacting AI implementation and sustained use in healthcare organisations. Rather than directly determining implementation decisions, these conditions create enabling or constraining environments through governance structures, investment, and skills development. Participants highlighted institutional mechanisms such as regulatory sandboxes and national health authorities that facilitate exposure to AI innovations while ensuring oversight. Conversely, fragmented or overlapping data governance regimes were seen to complicate interpretation and slow engagement.

Beyond governance, sustained public investment in digital infrastructure and AI capability development was identified as critical. National prioritisation of digital technologies, including funding initiatives and professional training, determines the availability of skills, tools, and resources at the organisational level. In this way, national digital conditions act as a form of scaffolding that influences the scope, pace, and scalability of AI implementation. Taken together, these findings suggest that national digital conditions operate as an institutional scaffolding that strengthens or constrains organisational readiness for AI implementation. Rather than directly driving implementation decisions, national digital scaffolding influences the organisational capabilities, resources, and governance environments through which AI implementation becomes feasible, scalable, and sustainable.

### 4.3 Technological factors

The findings indicate that technological factors constitute critical conditions influencing AI implementation and sustained use in healthcare not only through system performance, but through how AI systems generate, justify, and sustain clinical outputs over time. Figure 3 captures the Gioia data structure for technological factors shaping AI adoption.

**Figure 3:**
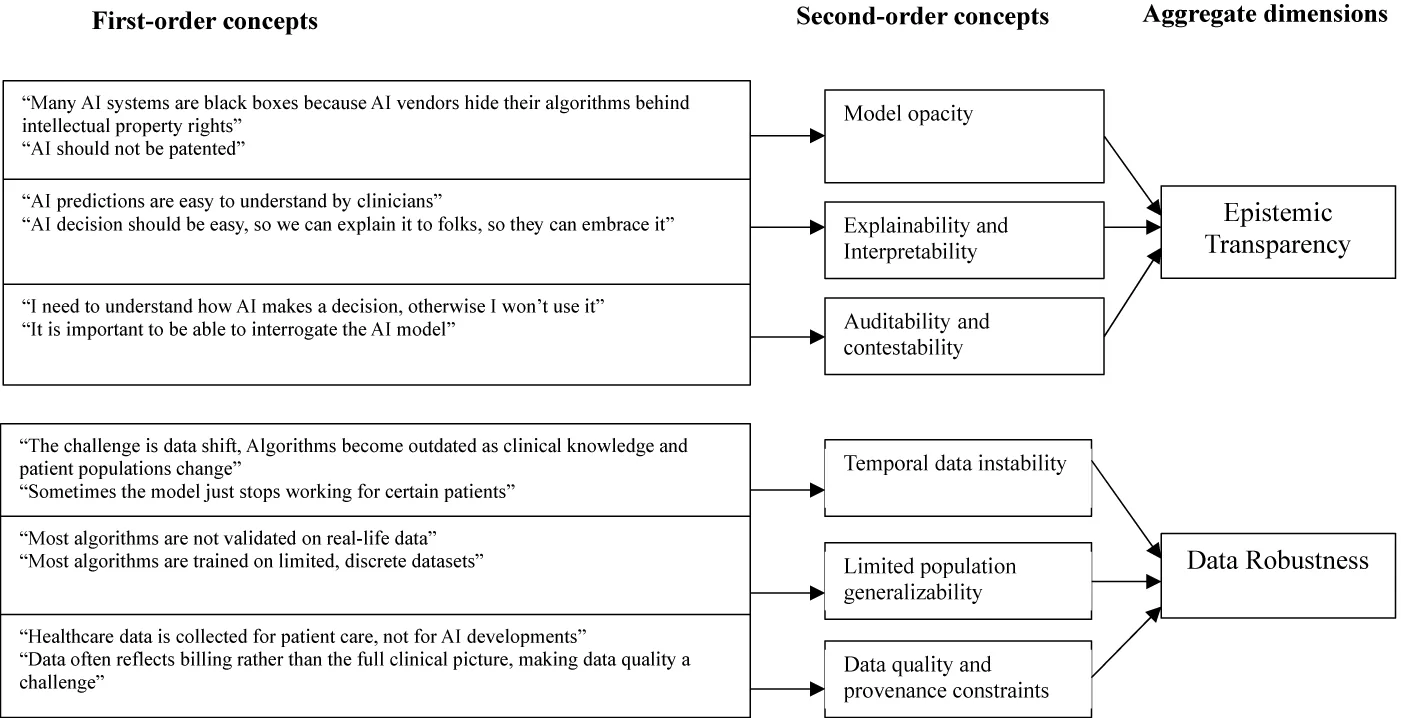
Gioia data structure: technological conditions shaping AI adoption. Source: Authors’ own work

#### 4.3.1 Epistemic transparency

Participants consistently framed model-related characteristics as central technological condition influencing whether AI systems could be trusted and meaningfully used in clinical practice. Rather than focusing solely on technical performance, they emphasised the importance of being able to understand, interrogate, and justify AI outputs in ways that align with clinical reasoning and professional accountability. Concerns about opaque model architectures, proprietary restrictions, and the inability to trace how predictions were generated were frequently described as undermining confidence in AI systems. In contrast, features that enabled explanation, interpretability, and post hoc interrogation were perceived as essential for integrating AI into clinical workflows and for sustaining trust among clinicians and patients. Collectively, these accounts indicate that participants evaluated AI systems not only according to their predictive accuracy but also according to the transparency of the knowledge claims they produced. Accordingly, this study conceptualises these characteristics as *epistemic transparency*, a technological condition through which explainability, interpretability, and contestability jointly influence organisational AI implementation. While explainability primarily concerns making AI outputs understandable, epistemic transparency refers more broadly to the extent to which AI-generated knowledge claims can be interrogated, justified, contested, and defended within clinical decision making and organisational governance (Floridi *et al.*, 2018; Grote and Berens, 2020; London, 2019).

#### 4.3.2 Data robustness

Participants' accounts pointed to data-related constraints as a central technological condition influencing AI implementation in healthcare, extending beyond issues of data availability to concerns about whether AI systems can sustain reliable and defensible performance across time, populations, and clinical settings. Participants repeatedly highlighted that changes in clinical knowledge and patient demographics undermine the temporal stability of AI models, while training on narrow or highly specialised datasets limits their applicability beyond the contexts in which they were developed. In addition, routine healthcare data collection practices, which are primarily designed for direct care rather than analytical reuse, were perceived as embedding quality, completeness, and provenance limitations that weaken AI performance. Taken together, these concerns extended beyond isolated data quality issues to reflect a broader organisational expectation that AI systems should remain dependable across changing clinical environments. Accordingly, this study conceptualises these interrelated concerns as *data robustness*, a technological condition that influences clinicians' confidence in AI systems and their willingness to integrate them into routine clinical decision making.

## 5. Interaction patterns shaping AI adoption in healthcare

Building on the thematic analysis, this section synthesises the findings by identifying recurring interaction patterns across technological, organisational, and environmental conditions influencing AI implementation decisions in healthcare organisations. Rather than representing isolated determinants, these patterns illustrate how AI implementation emerges through the dynamic interplay of multiple conditions operating simultaneously within organisational and institutional contexts.

The interaction patterns are derived from both participants' accounts and a second-stage abductive interpretive analysis. In some instances, participants explicitly described how one condition influenced or depended upon another. In other instances, the interaction patterns emerged through systematic cross-case comparison and interpretive analysis of recurring relationships across the dataset. Accordingly, the patterns should be understood as empirically grounded interpretive configurations rather than as relationships that were uniformly articulated by individual participants.

This interpretive approach is consistent with configurational qualitative inquiry, where higher-order explanations emerge through analytical synthesis of recurring relationships across cases rather than through isolated participant statements. Consequently, the interaction patterns represent context-dependent organisational regularities that explain how combinations of technological, organisational, and environmental conditions jointly influence AI implementation decisions.

While the preceding findings demonstrate that each dimension influences AI implementation, the second-stage analysis indicates that these influences rarely operate independently. Instead, AI implementation in healthcare emerges through recurring combinations of interacting conditions, whereby the significance of technological characteristics, organisational capabilities, and environmental contexts depends on their alignment, reinforcement, or tension within specific clinical and institutional settings. The five interaction patterns presented below illustrate these recurring configurations and collectively explain how AI adoption is shaped through the interaction of multiple organisational conditions rather than through the independent effects of individual determinants. The cross-case distribution and evidential basis of these five interaction patterns are summarised in Section 6.

### 5.1 Regulatory risk and technological defensibility in clinical contexts

A first interaction pattern concerns the relationship between risk allocation ambiguity and epistemic transparency and data robustness. This interaction was explicitly articulated by several participants, who linked regulatory uncertainty, professional accountability, and liability concerns to heightened demands for transparency, explainability, auditability, and robust data performance. First, we observed a recurring association between epistemic transparency and concerns about risk ambiguity.

A lot of AI software vendors hide behind intellectual property rights, leading to black box phenomena. In future, we should have regulations about AI transparency, explainability (R9).

AI Regulations should be developed to make sure that all models are being developed and deployed well (R2).

Across cases, participants consistently linked regulatory uncertainty with the need for greater technological defensibility. When legal responsibility remained unclear, clinicians expected AI systems to provide transparent reasoning, auditable decision processes, and evidence of robust performance to support defensible clinical judgement. Conversely, opaque models and concerns regarding data quality further amplified uncertainty surrounding professional accountability because clinicians found it more difficult to justify AI-assisted decisions.

Accordingly, this interaction pattern suggests that the influence of epistemic transparency and data robustness becomes particularly pronounced in healthcare environments characterised by elevated regulatory uncertainty and liability concerns. Rather than representing independent determinants, technological defensibility and regulatory risk reinforce one another, jointly shaping organisational willingness to adopt AI within clinical practice.

### 5.2 Institutional support and organisational mobilisation for AI in healthcare

A second interaction pattern highlights the relationship between national digital scaffolding and economic alignment and capability orchestration. Unlike the previous pattern, this interaction emerged primarily through cross-case comparison and interpretive analysis. Strong national governance arrangements, healthcare-specific digital infrastructure, and public investment in AI capabilities appear to enable healthcare organisations to commit financial resources and mobilise internal and external expertise.

The priority of this year within the national lab is education … implementing knowledge into curricula for doctors, nurses, therapists, and pharmacists because it is currently lacking (R19).

Funding is depending on adoption of technological innovation … in many hospitals, it is now necessary to adopt these innovations because otherwise there will be no money (R3).

These accounts suggest that institutional support extends beyond providing financial resources. National investment in digital infrastructure, education, and governance also legitimises organisational investment in AI, strengthens internal capability development, and facilitates collaboration with external partners. Consequently, organisations operating within supportive institutional environments are better positioned to align financial resources with strategic priorities and coordinate the capabilities necessary for successful AI implementation.

Conversely, where national support mechanisms are fragmented or underdeveloped, organisations experience greater difficulty securing investment, developing internal expertise, and sustaining AI initiatives, even when clinical interest and technological opportunities exist. This interaction pattern therefore suggests that organisational readiness for AI implementation is strongly conditioned by broader institutional arrangements that provide resources, legitimacy, and long-term strategic support for digital transformation.

### 5.3 Organisational capability and the sustainability of clinical AI systems

A third interaction pattern centres on capability orchestration and data robustness. This interaction was reflected in participant accounts emphasising that reliable AI performance depends not only on technical quality but also on organisational capabilities that support monitoring, evaluation, and continuous improvement. Healthcare organisations with stronger capability orchestration are better positioned to manage clinical data quality, monitor AI performance over time, and respond to challenges such as data drift, population heterogeneity, and changing care pathways. Where such capabilities are limited, data-related issues place additional operational and governance burdens on organisations and undermine confidence in the sustained clinical use of AI systems. This pattern highlights a reinforcing relationship in which organisational capacity supports the long-term viability of clinical AI, while technological fragility exposes organisational constraints.

In our hospital, we have a great environment, and we are very lucky because we have strong relationships and partnerships with major groups and vendors like Philips. I work with physicist from Philips to ensure that AI models are working well and how we can further refine them (R11).

AI companies should work with healthcare organisations, so they can build together their own AI, and not sell them a developed AI software to ensure that AI models act consistently across years (R2).

Participants described organisational capabilities such as multidisciplinary collaboration, external partnerships, continuous monitoring, and iterative model refinement as essential mechanisms for maintaining data quality and sustaining AI performance in changing clinical environments. These capabilities enabled organisations to respond proactively to challenges such as data drift, evolving patient populations, and changing clinical pathways.

Accordingly, this interaction pattern demonstrates that data robustness is not solely a technological property but is sustained through organisational capability. The long-term viability of clinical AI therefore depends on healthcare organisations' ability to continuously orchestrate expertise, partnerships, and evaluation mechanisms rather than relying exclusively on the technical performance of AI systems.

### 5.4 Economic incentives and the development of trust in healthcare AI

A fourth pattern concerns the interaction between economic alignment and epistemic transparency. This pattern emerged primarily through cross-case comparison and interpretive analysis rather than through uniformly explicit participant accounts. When funding structures, incentives, and leadership commitment support long-term AI use, healthcare organisations appear more willing to invest in transparency-enhancing practices, including explainability, validation, and ongoing clinical evaluation. In contrast, short-term or project-based funding arrangements tend to prioritise rapid deployment and limit tolerance for AI systems that require sustained interpretive effort from clinicians. This pattern suggests that economic conditions influence how trust in AI is developed and maintained within healthcare organisations.

We are going to invest in teaching basic informatics to all persons … [additionally] we have to find trustworthy AI partners to solve the issues of trust, black box (R5).

They are trying to sell me AI products saying that it will make your ROI return on investment high, […] during evaluation we could find that there are a lot of issues, mostly related to data. […] so, we have forum, which we call clinical data scientists form to discuss all these new tools (R9).

These accounts indicate that healthcare organisations do not develop trust in AI passively. Rather, trust appears to emerge through deliberate organisational investments in education, evaluation, governance mechanisms, and partnerships that enable AI systems to be scrutinised and understood. In this sense, economic alignment not only facilitates AI implementation but also creates the conditions through which epistemic transparency can be strengthened and sustained over time.

### 5.5 Environmental risk and strategic prioritisation of AI applications

A final interaction pattern involves risk allocation ambiguity and capability orchestration, particularly in relation to strategic prioritisation. Unlike some of the preceding patterns, this interaction was frequently articulated directly by participants when discussing how liability concerns influenced decisions regarding which AI applications should be prioritised, adopted, or avoided. Under conditions of high liability exposure and unclear accountability for AI-assisted clinical decisions, healthcare organisations tend to prioritise narrowly scoped or low-risk AI applications, such as administrative or decision-support tools with limited clinical autonomy. Adoption is more cautious in areas involving direct diagnostic or treatment decisions. This pattern indicates that environmental risk affects not only whether AI is adopted in healthcare, but also which types of AI applications are considered acceptable within existing organisational capabilities.

There's a lot of hesitation because of the dangers and also due to the fact that there's no regulation that spreads out the liability …. if the AI software will make decisions about life and death, I will be more hesitant, and I will check more and more (R9).

If you say it's clinical decision support that could help me, then I put it beside my opinion. I know what I want to do, meanwhile, I see well the recommendation of the AI (R5).

This interaction pattern shows that environmental risk affects not only the likelihood of AI adoption but also the strategic allocation of organisational capabilities across different categories of AI applications. AI systems that complement clinical judgement are prioritised because they align more closely with existing governance structures and organisational risk tolerance, whereas applications involving greater clinical autonomy are approached more cautiously under conditions of regulatory uncertainty and ambiguous liability.

## 6. Cross-case distribution and synthesis of interaction patterns

The five interaction patterns presented in the preceding section differed in both the breadth and form of their empirical support across the dataset. To make this evidential basis explicit, this section examines the cross-case distribution of the patterns across participants and institutional contexts. Table 2 identifies the participant accounts contributing substantive evidence to each interaction pattern, their contextual distribution, and whether the relationship was supported through direct participant articulation, cross-case interpretive analysis, or a combination of both. Consistent with the qualitative and abductive design of the study, these distributions are used to demonstrate the breadth and mode of empirical support rather than to indicate statistical prevalence or generalisability.

**Table 2:** Cross-case distribution and evidential basis of interaction patterns

Interaction pattern	Constituent dimensions	Supporting informants	Contextual distribution	Evidential basis
Regulatory risk and technological defensibility	Risk allocation ambiguity × epistemic transparency/data robustness	R2, R5, R8, R9, R18, R21, R25, R29	US, France, Netherlands, Singapore, Switzerland, Belgium	Direct + cross-case
Institutional support and organisational mobilisation	National digital scaffolding × economic alignment/capability orchestration	R1, R3, R9, R18, R19, R22, R25, R26, R27, R28	US, Netherlands, Singapore, Switzerland, UK, Estonia	Primarily cross-case
Organisational capability and sustainability of clinical AI	Capability orchestration × data robustness	R1, R2, R7, R8, R9, R10, R11, R12, R19, R22, R28, R29	US, France, Netherlands, Singapore, Estonia, Belgium	Direct + cross-case
Economic incentives and development of trust	Economic alignment × epistemic transparency	R3, R5, R9, R22	US, Singapore	Primarily cross-case
Environmental risk and strategic prioritisation	Risk allocation ambiguity × capability orchestration	R3, R5, R9, R18, R21, R22, R25	US, Netherlands, Singapore, Switzerland	Predominantly direct + cross-case

**Note(s):** Direct support refers to explicitly articulated relationships, whereas cross-case support reflects relationships identified through comparative abductive analysis. Informant distributions indicate evidential breadth, not statistical prevalence

**Source(s):** Authors’ own work

The cross-case distribution indicates variation in both the breadth and form of empirical support across the five interaction patterns. Patterns 1 and 3 showed comparatively broad support across participants and institutional contexts, combining explicitly articulated relationships with recurring cross-case evidence. Pattern 5 was also supported by direct accounts linking liability and accountability uncertainty to more cautious approaches towards AI applications involving greater clinical autonomy. Pattern 2 was distributed across several contexts but emerged more strongly through cross-case comparison of institutional and organisational conditions. Pattern 4 had a comparatively narrower evidential base and was primarily interpretive, reflecting a cross-case relationship between economic alignment and epistemic transparency rather than one consistently articulated within individual participant accounts. These differences should not be interpreted as measures of statistical prevalence or relative importance, but as variation in the breadth and mode of empirical support for the identified configurations.

Taken together, the cross-case analysis demonstrates the value of moving beyond an independent-determinants account of AI adoption. While transparency, data robustness, organisational capability, economic alignment, regulation, and institutional support emerged as relevant conditions, their implications depended on how they combined. For example, transparency and robustness became more consequential under liability uncertainty, while sustained technical robustness depended partly on organisational capabilities for monitoring and adaptation. Similarly, regulatory risk influenced both implementation decisions and the level of clinical autonomy organisations were prepared to accept. The interaction-pattern analysis therefore shows how the same condition can have different implications depending on the wider organisational and environmental configuration in which it occurs.

## 7. Discussion

The findings support the central proposition of configurational scholarship that organisational outcomes emerge through combinations of interacting conditions rather than the independent influence of individual factors (Ragin, 2008). While prior research often examines elements such as explainability, organisational readiness, or regulatory pressure in isolation, the results show that their influence depends on how they align with one another. AI adoption is therefore better understood as a context-dependent process emerging from configurations of conditions rather than as a linear, factor-driven decision. For instance, the relationship between risk allocation ambiguity and epistemic transparency becomes particularly influential under conditions of regulatory uncertainty, whereas the interaction between capability orchestration and data robustness depends upon organisations' ability to mobilise expertise, resources, and collaborative partnerships to sustain AI performance over time. These findings further suggest that the identified interaction patterns are activated under different organisational and institutional conditions rather than operating uniformly across healthcare settings. Consequently, the significance of any individual technological, organisational, or environmental condition depends on the broader configuration within which it is embedded, helping to explain why similar AI technologies may experience markedly different adoption trajectories across healthcare organisations.

The findings also extend process-relational perspectives (Orlikowski, 2000) and Actor-Network Theory by suggesting that AI adoption is not solely determined by technological characteristics or organisational readiness but is continually negotiated through interactions among clinicians, organisational leaders, AI developers, regulators, and AI systems themselves. The identified interaction patterns illustrate that AI adoption is shaped through ongoing negotiations concerning accountability, legitimacy, governance, and organisational capability. Rather than viewing AI technologies as passive organisational tools, the findings highlight how technological artefacts, organisational actors, and institutional arrangements collectively influence adoption trajectories within healthcare organisations. This perspective reinforces the view that AI adoption can be better understood as an evolving organisational process rather than a discrete implementation decision.

Within the healthcare domain, the findings extend insights that emerged from Non-adoption, Abandonment, Scale-up, Spread, and Sustainability (NASSS) framework (Greenhalgh *et al.*, 2017). While the latter shows the interaction complexity between specific domains, this study shows how specific interactions and configurations can lead to different adoption trajectories. This perspective helps explain inconsistencies in the AI adoption literature, particularly in studies using determinant-oriented frameworks such as TOE, TAM, and UTAUT, which report mixed findings across healthcare settings (Venkatesh *et al.*, 2003; Baker, 2012). It also aligns with qualitative and process-oriented research showing that digital innovation outcomes are conditioned by institutional context, professional norms, and governance arrangements (Greenhalgh *et al.*, 2017). By foregrounding interaction patterns, this study extends prior work by demonstrating how these conditions jointly influence adoption under regulatory ambiguity, organisational constraints, and technological uncertainty.

The findings further resonate with recent healthcare AI research emphasising governance capacity, accountability, and organisational capability as central to sustainable adoption. Attributes such as explainability, trustworthiness, and robustness are not inherent to AI systems but are realised through organisational processes and institutional support (Reddy *et al.*, 2020; Lekadir *et al.*, 2025). The observed pattern of selective adoption, where organisations prioritise lower-risk applications under uncertainty, is consistent with evidence that healthcare organisations adopt complex technologies incrementally in response to accountability and patient safety concerns (Alami *et al.*, 2020; Greenhalgh *et al.*, 2017). Overall, these findings reinforce the importance of interaction-sensitive explanations for understanding AI adoption in healthcare and indicate that examining recurring configurations of conditions provides additional explanatory insight beyond considering individual determinants independently.

## 8. Theoretical contributions

This study contributes to the healthcare AI adoption literature by extending configurational thinking within the TOE framework through the identification of recurring interaction patterns among technological, organisational, or environmental determinants. The findings suggest that the explanatory value of the TOE framework can be strengthened by considering how its technological, organisational, and environmental domains operate configurationally. Drawing on abductive evidence, the findings suggest that the explanatory relevance of these domains is relational and conditional. Technological, organisational, and environmental factors do not exert stable effects across contexts, instead, their relevance emerges through patterned interactions that inform how adoption decisions are evaluated and legitimised. By shifting the analytical emphasis from independent determinants to recurring interaction patterns, the study extends configurational perspectives on organisational technology adoption and provides a more context-sensitive explanation of AI adoption within the institutional complexity of healthcare organisations (Orlikowski, 2000; Langley *et al.*, 2013).

A second theoretical contribution concerns the role of technological characteristics in organisational AI adoption. Prior research often treats characteristics such as transparency, robustness, and explainability as intrinsic properties of AI systems that directly influence adoption (Reddy *et al.*, 2020). The present findings suggest a different interpretation. As demonstrated by the cross-case synthesis in Section 6, these conditions did not operate uniformly or independently across cases. Rather than exerting universal effects, these technological characteristics appear to acquire organisational significance through their interaction with capability orchestration, governance arrangements, and accountability structures. Consequently, the value of technological attributes is not fixed but contextually activated by the organisational and environmental conditions within which AI systems are implemented. This perspective provides a more nuanced explanation of why similar AI technologies may be adopted, constrained, or deferred across healthcare organisations facing different institutional configurations.

Finally, the study contributes to the broader organisational adoption literature by providing an empirically grounded account of how interaction patterns help explain variation in AI adoption decisions across healthcare settings. Rather than proposing a new adoption theory, the findings refine existing TOE-based explanations by demonstrating that organisational adoption can be more fully understood through recurring configurations of interacting conditions. In doing so, the study bridges determinant-based technology adoption research with configurational and process-relational perspectives, offering more context-sensitive theoretical explanation of how AI adoption decisions are formed, negotiated, and enacted in highly regulated organisational environments.

## 9. Practical implications

The findings suggest that optimising AI implementation within healthcare organisations cannot be achieved by addressing technological, environmental, and organisational conditions in isolation. Rather, the study suggests that different combinations of these conditions can create different adoption situations that require different managerial responses. Therefore, the practical value of the five identified interaction patterns does not lie in identifying single implementation pathway, but in enabling organisations to distinguish between conditions that managers can actively influence, and those they must navigate and adapt to.

The first interaction pattern suggests that healthcare organisations should not address regulatory uncertainty, data robustness and epistemic transparency as distinct implementation challenges. When legal responsibility for AI-assisted clinical models remains not clearly defined and structured, managers can bolster organisational readiness by prioritising AI models that offer explainability, transparency, auditability, and robust clinical validation. In other words, creating internal governance structures, such as those that assess whether AI systems demonstrate these features, alongside documenting of AI outputs and regularly evaluating model performance are practical actions that could reduce physicians' concerns about the lack of risk allocation framework. However, healthcare organisations cannot address the broader structural constraints like national liability regimes through managerial actions alone instead, they have to adapt their implementation strategies to the existing regulatory environment, while healthcare policy makers must continue elaborating AI responsibility frameworks. Therefore, this interaction patterns suggests that managers should focus on improving technological defensibility to maintain balance amidst broader regulatory uncertainty.

The second interaction pattern suggests that internal commitment and engagement alone are not sufficient for AI implementation within healthcare organisations. They also require support from the institutional environment where they function. The findings indicate healthcare managers can facilitate AI implementation by aligning AI investments with strategic priorities, developing multidisciplinary AI capabilities, investing in staff training, and fostering partnerships with academic institutions and technology providers. By undertaking these actions, healthcare organisations have to mobilise the financial, technical, and clinical resources needed for a successful implementation of AI. At the same time, the findings indicate that providing these resources require broader institutional support, including national investment in digital infrastructure, healthcare-specific AI education, dedicated funding programmes, and robust governance frameworks. Without, or with only limited, institutional support, organisations are likely to face significant challenges in securing long-term investment, developing specialist expertise, and sustaining AI initiatives. Therefore, while policymakers are responsible for creating appropriate institutional conditions through investments, governance frameworks, and supportive policies; healthcare managers are responsible for mobilising and effectively deploying these capabilities within their own organisations.

The third pattern suggests that the sustained use of AI depends on organisational capabilities as well as technical quality. Healthcare managers may strengthen the conditions supporting data robustness by investing in multidisciplinary collaboration, developing external technical partnerships, and establishing governance structures for continuous monitoring, evaluation, and model refinement. These represent actionable organisational leverage points. By contrast, changes in patient populations, data distributions, and clinical pathways are partly structural features of healthcare environments that organisations cannot eliminate but can seek to accommodate through monitoring and adaptive governance.

The fourth pattern suggests that organisational investment may support the conditions under which trust in clinical AI can develop. Resources allocated to education, AI evaluation, validation, and partnerships can strengthen organisations' capacity to scrutinise AI systems and improve epistemic transparency. However, these activities require sustained financial and organisational commitment and may be difficult to maintain under short-term or project-based funding arrangements. Accordingly, longer-term investment represents a potential managerial leverage point, while unstable external funding structures may constitute a constraint that individual organisations have limited capacity to change.

The fifth interaction pattern suggests that, under conditions of unclear liability and regulatory uncertainty, healthcare managers may reduce implementation risk by prioritising AI applications that support rather than replace clinical judgement, particularly lower-risk applications with limited clinical autonomy. In such contexts, a phased implementation approach may provide a practical means of aligning organisational readiness, application risk, and the prevailing regulatory environment.

Taken together, the five interaction patterns distinguish between conditions that are potentially amenable to managerial intervention and those that largely constitute structural constraints. Potential leverage points include governance arrangements, resource allocation, multidisciplinary capability development, evaluation practices, and clinician engagement. By contrast, regulatory uncertainty, liability structures, national digital infrastructure, and some external funding arrangements are conditions that individual healthcare organisations have limited capacity to alter and must therefore largely accommodate. Given the exploratory qualitative design and composition of the sample, these implications should be interpreted as empirically informed considerations for organisational decision-making rather than universally applicable prescriptions.

## 10. Limitations and future research

Several limitations should be considered when interpreting the findings of this study. First, the data collection period (2021–2025) coincided with substantial technological, regulatory, and institutional developments in the broader AI landscape. Although the year-by-year comparative analysis indicated that the six aggregate dimensions and five interaction patterns remained analytically stable across the study period, evolving perceptions of AI may nevertheless have influenced how participants discussed organisational adoption. Future longitudinal research could examine whether these interaction patterns remain stable as AI technologies and regulatory environments continue to evolve.

Second, although participants were drawn from multiple healthcare systems, the sample was concentrated in the United States and France, with several countries represented by only one or two participants. Consequently, the study was not designed to identify country-specific adoption patterns or support cross-national comparisons. Instead, the findings should be interpreted as analytically generalisable interaction patterns that recur across diverse healthcare contexts rather than as evidence of systematic national differences. Future studies could examine how these interaction patterns vary across specific healthcare systems, regulatory environments, or cultural contexts.

Third, theoretical saturation was achieved at the level of the second-order themes and aggregate dimensions rather than across individual countries, healthcare organisations, or participant groups. Although later interviews consistently reinforced the existing conceptual structure, context-specific organisational mechanisms may remain underexplored. Future qualitative and comparative studies could investigate how the identified interaction patterns manifest within organisational settings, clinical specialties, or national healthcare systems.

Fourth, the study relied primarily on senior clinicians, organisational leaders, policymakers, and AI specialists who were directly involved in AI adoption and governance. While these participants provided rich strategic and organisational insights, the perspectives of frontline clinicians, nurses, allied health professionals, and operational staff were less represented. Future research should incorporate these stakeholder groups to develop a more comprehensive understanding of how interaction patterns influence AI adoption throughout the implementation process.

Finally, although several participants held hybrid roles spanning healthcare organisations and health technology companies, the analysis prioritised their accounts relating to organisational implementation, governance, and healthcare decision making. Nevertheless, commercial perspectives may have indirectly influenced certain observations despite the analytical procedures adopted to minimise this possibility. Future research could explicitly compare organisational and vendor perspectives to further examine how different stakeholder positions influence perceptions of AI adoption.

Despite these limitations, the study provides an empirically grounded explanation of how technological, organisational, and environmental conditions interact to inform AI adoption decisions within healthcare organisations. By identifying recurring interaction patterns across diverse organisational contexts, the findings extend existing TOE-based explanations and provide a foundation for future research adopting configurational, longitudinal, and comparative approaches to organisational AI adoption.

## 11. Conclusion

This study examined how technological, organisational, and environmental conditions interact to inform AI adoption decisions in healthcare organisations. Drawing on an abductive qualitative analysis of healthcare experts' experiences, the findings suggest that organisational AI adoption can be understood as a process emerging through recurring interactions among technological, organisational, and environmental conditions, rather than solely through the independent influence of isolated determinants. By identifying six higher-order dimensions and five recurring interaction patterns, the study provides an empirically grounded explanation of how organisational AI adoption unfolds within the complex regulatory, professional, and institutional environment of healthcare.

The findings extend Technology-Organisation-Environment (TOE) explanations by suggesting that the influence of technological, organisational, and environmental conditions is inherently relational and context dependent. Rather than acting as universal drivers of adoption, these conditions acquire organisational significance through their interaction with one another, thereby providing a richer explanation of why similar AI technologies are adopted, constrained, or deferred across different healthcare organisations.

From a practical perspective, the study highlights that responsible and sustainable AI adoption requires more than technological excellence alone. Successful implementation depends on aligning technological defensibility, organisational capabilities, and supportive institutional environments through appropriate governance, capability development, and strategic investment. Collectively, these findings provide a more comprehensive foundation for future research, organisational decision making, and policy development aimed at supporting the responsible adoption of AI within healthcare systems.

## Declaration on the use of generative AI

During the preparation and revision of this manuscript, the authors used ChatGPT solely for copy-editing of author-created text, including proofreading and refinement of language, grammar, clarity, and readability. Generative AI was not used to create or generate the research questions, theoretical framework, research design, research data, data analysis, findings, interpretations, or conclusions. All substantive intellectual content, concepts, arguments, analyses, and interpretations were developed by the authors.
